# Does Bariatric Surgery Affect Pregnancy in Women in the Childbearing Period? A Retrospective Single Center Study

**DOI:** 10.1007/s11695-025-08197-6

**Published:** 2025-08-29

**Authors:** Mohamed Alsharkawy, Mohamed Baklola, Mohamed Terra, Amr Sanad, Ali Salem, Mohamed El Sorogy, Amgad Fouad, Ahmed Abdelrafee

**Affiliations:** Mansoura University, Al Mansurah, Egypt

**Keywords:** Bariatric surgery, Pregnancy, Childbearing, Infertility

## Abstract

**Background:**

Severe obesity is a significant public health challenge, affecting women’s reproductive health in the childbearing period. Bariatric surgery is an effective weight loss method for management of severe obesity and increase fertility. It may result in nutritional deficiency which adversely affects the maternal and fetal outcome.

**Methods:**

This was a retrospective study included all women with severe obesity in the childbearing period who underwent bariatric surgery in the period between March 2011 and June 2023. The study cohort was divided into three groups according to fertility status: fertile, infertile, and potential fertility status (unmarried). Fertility rate and pregnancy outcome data were evaluated according to the interval between the bariatric surgery and conception.

**Results:**

Out of 177 women included in the study cohort, 134 women (75.7%) were fertile, 16 (9%) were with infertility, 27 (15.3%) were unmarried. The incidence of PCOS was 13.6%. Overall rate of conception was 28.8% mostly in the women with infertility group (*n* = 12, 75%) with median interval 20 months from surgery. Pregnancy complications were encountered in 36 women (70.6%), among of which 13 women (86.7%) got pregnant after 2 years from surgery. Anemia was the most common complication (*n* = 28, 54.9%), followed by hemorrhage (*n* = 20, 39.2%). Ten neonates (30.3%) were small for gestational age in women with completed pregnancy and no congenital anomalies occurred.

**Conclusion:**

Bariatric surgery may improve the fertility rates. However, pregnancies after surgery may result in potential complications particularly if the pregnancy occurs early during the first year and delayed after 2 years from surgery. Lack of adequate multidisciplinary follow-up and specific prenatal program for women after bariatric surgery increases the risk of gestational complications and unfavorable pregnancy outcomes.

**Graphical Abstract:**

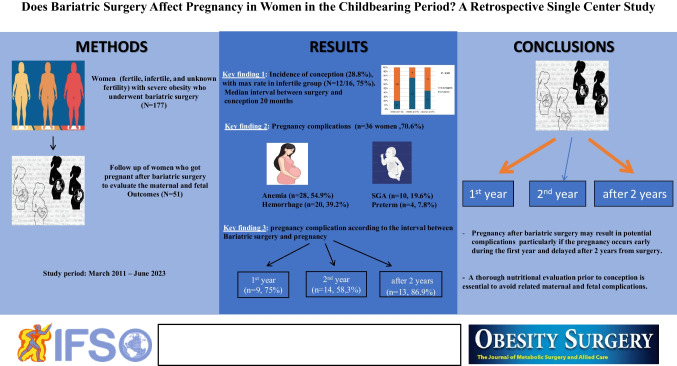

**Supplementary Information:**

The online version contains supplementary material available at 10.1007/s11695-025-08197-6.

## Background

Obesity is a significant public health challenge, with its prevalence steadily increasing. It is associated with medical problems that significantly impair health outcomes, including reproductive health [1]. For women in the childbearing period, obesity is linked to menstrual dysfunction, ovulatory disorders, and conditions such as polycystic ovarian syndrome (PCOS), three folds more than women with no obesity, which directly impact fertility [2]. Moreover, childhood obesity is associated with increased risk of female infertility in the reproductive-age life [3]. Additionally, obesity contributes to complications during pregnancy, adversely affecting both maternal and fetal outcomes, including an elevated risk of gestational diabetes, preeclampsia, and neonatal complications [4]. Moreover, obesity increases the likelihood of long-term health challenges for offspring, such as metabolic syndrome, underscoring the intergenerational impact of this condition [2, 4].

The pathophysiology underlying obesity-related reproductive dysfunction is complex, involving metabolic, hormonal, and inflammatory pathways [5]. Obesity is characterized by insulin resistance, hyperinsulinemia, and increased androgen levels, which impair ovarian function. PCOS, a common condition in obese women, is marked by anovulation, menstrual irregularities, and hyperandrogenism, contributing to subfertility [6, 7]. Additionally, obesity is associated with increased levels of inflammatory cytokines and altered adipokine profiles, which can disrupt the hypothalamic-pituitary-ovarian axis [7].

Bariatric surgery, encompassing procedures such as Roux-en-Y gastric bypass (RYGB), sleeve gastrectomy, and adjustable gastric banding, has emerged as an effective intervention for severe obesity [8]. These procedures induce significant weight loss through humoral mechanisms, mainly of incretin origin [8]. Beyond weight reduction, bariatric surgery has demonstrated a major impact on reproductive health [9]. Studies indicated that post-surgical weight loss can normalize hormonal imbalances, leading to improved ovulation rates, regularized menstrual cycles, and enhanced fertility [10]. For women with obesity-related infertility, bariatric surgery offers a potential pathway to conception when traditional weight-loss strategies and medical treatments fail [11].

Despite the benefits of bariatric surgery, the timing of conception post-surgery remains a critical consideration [12]. Rapid weight loss following surgery, coupled with potential micronutrient deficiencies, poses risks to maternal and fetal health if pregnancy occurs too soon [12]. Clinical guidelines recommend delaying pregnancy for 12–24 months post-surgery to allow for weight stabilization and nutritional optimization [13]. During this period, patients typically experience substantial improvements in metabolic health, including reductions in insulin resistance, dyslipidemia, and hypertension, which are vital for favorable pregnancy outcomes [13].

Regarding to neonatal outcome, post-bariatric surgery pregnancies have potential risks affecting the maternal and fetal health [4]. Studies highlight a potential increased incidence of small-for-gestational-age (SGA) infants, preterm birth, and nutritional deficiencies in neonates born to mothers who conceive after bariatric surgery [14, 15]. These risks underscore the importance of preconception counseling, meticulous nutritional monitoring, and individualized care plans for this population [16].

Current evidence emphasizes the need for further research to better understand the relationship between bariatric surgery and reproductive health [8, 13]. Additionally, there is limited data specific to women of reproductive age undergoing bariatric surgery in low- and middle-income countries, such as Egypt, where healthcare infrastructure and follow-up protocols may differ significantly from those in high-income settings [17]. This highlights the need for region-specific studies to address unique challenges and improve outcomes for women in these contexts.

This study aims to address these knowledge gaps by examining the impact of bariatric surgery on fertility and pregnancy outcomes in a cohort of women with severe obesity in the childbearing period.

## Patient and Methods

### Study Design and Population

This retrospective cohort study was conducted including all women with severe obesity aged 18 to 45 in their childbearing period who underwent bariatric surgery between March 2011 and June 2023.

### Inclusion Criteria

Women aged 18 to 45 years old who underwent different types of bariatric surgeries in the study period were included in the study. Indications of bariatric surgery are severe obesity with body mass index (BMI) ≥ 40 kg/m^2^ and BMI from 35 to 40 kg/m^2^ with associated medical problems. The study enrolled women with primary and secondary infertility after bariatric surgery.

### Exclusion Criteria

Women were excluded if they had undergone menopause before operation, or had irreversible infertility status prior to surgery such as women with primary ovarian insufficiency or underwent hysterectomy and/or bilateral oophorectomy before bariatric surgery. The study precluded women who experienced surgical termination of pregnancy. Women who lost follow up after surgery or with missed data regarding the course and outcome of pregnancy were excluded from the analysis.

### Study Outcomes

Primary outcome of the study was the incidence of pregnancy in fertile women and women with infertility associated with severe obesity after different types of bariatric surgery. The secondary outcomes included the effect of the interval between the surgery and the conception on the pregnancy outcomes, the perinatal outcomes regarding maternal complications such as gestational diabetes and hypertension and fetal complications and the need of neonatal intensive care (NICU). In addition, the effect of bariatric surgery on the associated comorbidities and weight loss was evaluated.

The study cohort was stratified into three groups based on their fertility status before the surgery. The first group consisted of women with infertility which had potentially reversible causes such as ovulation disorder (eg.PCOS), uterine fibroid, endometriosis, fallopian tube blockage, and certain medical disease like hypothyroidism. The second group included women without reported fertility issues prior to the surgery. The third group consisted of women who were potentially fertile because they were unmarried and the sexual relationships prior to marriage are prohibited.

### Data Collection

Data were extracted from the institution’s web-based registry and supplemented with archived medical records. Missing follow-up data and pregnancy-related data were obtained by directly contacting the participants through telephone interviews.

Preoperative data included demographic and clinical variables, such as age at the date of surgery, BMI, marital and fertility status, presence of associated medical problems to severe obesity such as diabetes mellitus (DM), hypertension (HTN), obstructive sleep apnea (OSA), Gastro-esophageal reflux disease (GERD), and polycystic ovary syndrome (PCOS).

Intraoperative and postoperative data comprised details about the type of bariatric surgery performed, including common procedures such as gastric bypass, sleeve gastrectomy and gastric plication, possible early postoperative complications, and hospital stay.

### Follow Up Data

Follow up data included weight changes either degree of weight loss and incidence of weight regain, the improvement of preoperative medical problems, and manifestations of nutritional deficiencies such as hair loss and anemia.

In the group of women who conceived after bariatric surgery, retrieved pregnancy-related data included the occurrence of pregnancy after surgery, the interval between surgery and pregnancy and weight gain during pregnancy. There is no available specific follow up program for the women who got pregnant after bariatric surgery. IVF data included success rates and outcomes. Complications during pregnancy were divided into maternal and neonatal complications. Maternal complications included anemia, gestational diabetes mellitus (GDM), gestational hypertension (HTN), hemorrhage (e.g., antepartum hemorrhage), and possible nutritional deficiency. Neonatal complications included abnormal birth weight (appropriate for gestational age or small for gestational age) are recorded, as well as neonatal intensive care unit (NICU) admissions. Pregnancy outcome was subdivided into two groups, Group 1: pregnancy ended with miscarriage (abortion or stillbirth), Group 2: pregnancy completed to the time of delivery. Complications in each group were evaluated.

According to the severity, obstetric complications were classified into: Major and minor complications. Major complications are life-threatening and require immediate medical or surgical interventions such as GDM, miscarriage, hemorrhage, and preterm labour. Minor complications are less severe but still cause discomfort and require treatment such as anemia and gestational (HTN). Mode of delivery either vaginal or cesarean section was evaluated. The incidence of miscarriage (abortion and stillbirth) was recorded. In the group of women who did not conceive following surgery, causes of no pregnancy were investigated.

### Definitions

Infertility was defined and categorized into two subtypes for this study. Primary infertility refers to the inability to achieve pregnancy after 12 months or more of regular unprotected sexual relationship without a prior history of conception. Secondary infertility refers to the inability to conceive following a previous pregnancy despite regular unprotected intercourse for 12 months or more [1]. For analysis, both primary and secondary infertility were grouped under the broader category of infertility. In-vitro fertilization (IVF) success was defined as the fertilization and implantation of an embryo, irrespective of the pregnancy outcome [2]. Stillbirth was classified as fetal death occurring at or after 20 weeks of gestation, while abortion was defined as the loss of pregnancy before 20 weeks [3].

Total body weight loss (TWL) and percent TWL (%TWL) were used to express the weight loss data [18]. TWL is calculated as the ratio of the difference between the initial weight and postoperative weight to the initial weight. According to the recent IFSO consensus on definitions and clinical practice guidelines for obesity management, late postoperative clinical deterioration defined as recurrent weight gain or worsening the obesity complication after initial adequate clinical response. Recurrent weight gain is defined as the weight regain of ≥ 20% of the maximum total weight loss achieved (nadir) after surgery [19].

Gestational diabetes mellitus (GDM) was diagnosed using glucose tolerance testing during pregnancy [8], and gestational hypertension (HTN) was characterized by elevated blood pressure during pregnancy without preeclampsia [9]. Anemia during pregnancy was defined according to the trimester of pregnancy as the hemoglobin level is below 11, 10.5, and 11 g/dL in the first, second, and the third trimester respectively [20]. Early hemorrhage during pregnancy (EHG), was defined as blood loss occurring early in gestation [10], and antepartum hemorrhage (APH), was defined as vaginal bleeding occurring after 20 weeks of gestation and before delivery, were recorded [11]. Neonatal outcomes, categorized by gestational age, included preterm neonates born before 37 weeks of gestation [12], and at-term neonates delivered between 37 and 42 weeks [13]. Neonatal weight outcomes were classified as appropriate for gestational age (AGA), defined as birth weight between the 10th and 90th percentiles for gestational age [14], or small for gestational age (SGA), defined as birth weight below the 10th percentile [14, 15].

### Statistical Analysis

The data were coded and analyzed using SPSS software, version 25. Continuous variables were assessed for normality using the Kolmogorov–Smirnov and Shapiro–Wilk tests. Normally distributed continuous variables were summarized as means with standard deviations, while non-normally distributed variables were presented as medians and ranges. Categorical variables were expressed as frequencies and percentages. Comparisons of continuous variables were conducted using independent t-tests for normally distributed data, while the Mann–Whitney U test was employed for nonparametric data. Chi-square tests were applied for categorical variables, and Pearson’s correlation coefficient was used to explore relationships between continuous variables. A p-value of less than 0.05 was considered statistically significant.

## Results

### Preoperative Data of the Patients

The study included 177 patients, with a median age of 35 years (range: 18–45 years) and a median BMI of 49.8 kg/m^2^ (range: 33.6–87.6 kg/m^2^). The majority of patients were married (*n* = 147, 83%), followed by those who were single (*n* = 27, 15.3%) and a small proportion who were widowed or divorced (*n* = 3, 1.7%). Regarding fertility status, 75.7% of patients were fertile, 9% were infertile, and 15.3% had unknown fertility status. The median number of parities before surgery was 2 (range: 0–5).

Regarding the associated medical problems to obesity severe obesity, the most common was osteoarthritis (*n* = 103, 58.2%). Among patients with DM (*n* = 22, 12.4%), the median age of onset of DM was 29 years (range: 14–40 years), the median number of drugs used for treatment was 3 (range: 1–3), and 13 patients used oral hypoglycemic drugs, while the remaining 9 patients used insulin. Other common co-morbidities included hypertension (HTN) (*n* = 40, 22.6%), obstructive sleep apnea (OSAS) (*n* = 49, 27.7%), gastro esophageal reflux disease (GERD) (*n* = 40, 22.6%), and polycystic ovary syndrome (PCOS) (*n* = 24, 13.6%) as shown in Table 1.

**Table 1:** Periperative data of the patients

Variables	Number (Total number of patients = 177)	Percentage (%)
Age*	35 (18—45)	
Body Mass Index (BMI)*	49.8 (33.6—87.6)	
Marital status (before the operation)		
Single	27	15.3
Married	147	83
Others (Widow-Divorced)	3	1.7
Fertility Status		
Fertile	134	75.7
Infertile	16	9
Potentially fertile	27	15.3
Number of parities before the operation*	2 (0–5)	
Comorbidities		
Polycystic Ovary Syndrome (PCOS)	24	13.6
Diabetes Mellitus	22	12.4
Hypertension	40	22.6
Obstructive Sleep Apnea (OSAS)	49	27.7
Osteoarthritis	103	58.2
Hypothyroidism	6	3.4
Gastro Esophageal Reflux Disease (GERD)	40	22.6
Dyslipidemia	47	26.6
Type of operation:		
- Sleeve Gastrectomy	147	83.1
- Gastric Bypass	22	12.4
- SASJ	7	4
- Plication	1	0.6
Operation time (minutes)*	120 (40—270)	
Intra-operative complications	4	2.3
Post-operative internal haemorrhage	3	1.7
Hospital stay (days)*	3 (1—10)	

NB: * all variables expressed as median (min – max). SASJ: Single Anastomosis Sleeve Jejunal Bypass

### Operative and Early Postoperative Data

The majority of patients underwent sleeve gastrectomy (*n* = 147, 83.1%), with a smaller proportion undergoing gastric bypass (*n* = 22, 12.4%) including one anastomosis gastric bypass (*n* = 15) and Roux en-Y gastric bypass (*n* = 7), single anastomosis sleeve jejunal bypass (SASJ) (*n* = 7, 4%), and one case underwent gastric plication. Four patients (2.3%) experienced intraoperative complications. These included short gastric vessels bleeding in 2 patients controlled by clipping and/or suturing, stapler misfiring (*n* = 1), injury of the left liver lobe (*n* = 1) controlled by coagulation. Notably, there was no need for blood transfusion in any patient (Table 1).

Postoperative complications included internal hemorrhage in 3 patients (1.7%), with sources from port site, staple line, and short gastric vessels, and occurred in a median duration of 1 day (range: 1–6 days) after surgery. All cases were managed by laparoscopic exploration and control the source of bleeding. The median time for oral intake was 1 day (range: 1–5 days). There were no reported cases of anastomotic leakage.

### Follow-Up Data over 3, 6, and 12 Months

The median % TWL at the end of the first year was 32.8% (range: 17–54%). For recurrent weight gain, 49 patients (27.7%) regained weight ≥ 20% the maximum weight lost after operation. The percentage of occurrence of anemia was gradually increasing from 43.5% of patients by 3 months up to 45.2% of patients by 12 months. There was hair loss in 89.8% of patients and 26% of patients had de-novo reflux symptoms after surgery.

Regarding the associated medical problems, there was resolution of the majority of patients with DM, HTN, OSAS, and GERD at 3 months (72.7%, 80%, 75.5%, and 55%, respectively) For DM, 72.7% of cases resolved by 3 months. Osteoarthritis (joint pain) took longer time to be resolved in nearly half of the patients (*n* = 57, 55.3%) at 1 year after surgery, while 14 patients (13.6%) with advanced grade of osteoarthritis remained unchanged.

## Pregnancy Outcomes after Bariatric Surgery

### The Incidence of Conception

**Fig. 1:**
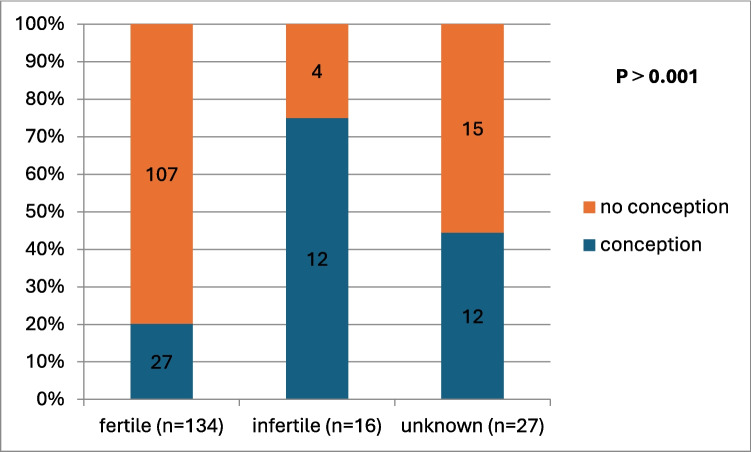
Conception rate following bariatric surgery according to the fertility status (fertile, infertile and potentially fertile (unknown)

Of the 177 patients, 51 (28.8%) reported pregnancies after bariatric surgery, of which 49 pregnancies were spontaneous and 2 were induced by IVF (Table 2). Miscarriage was encountered in 18 pregnant women including abortion (*n* = 17) and stillbirth (*n* = 1). All cases of abortion were preceded by hemorrhage either early hemorrhage (*n* = 15) or antepartum hemorrhage (*n* = 2). There was no any voluntary interruption of pregnancy. The incidence of conception was significantly higher in women with infertility group (12 out of 16, 75%), among of them 11 women had PCOS before surgery, and unfortunately 5 women (41.7%) had abortion as shown in Fig. 1. One hundred and twenty-six women did not conceive after surgery for the following reasons: contraception use (*n* = 79, 44.6%), still unmarried (*n* = 16, 9%), male infertility causes (*n* = 14, 7.9%), menopause (*n* = 7, 3.9%), uterine causes (*n* = 8, 4.5%), and ovarian causes (*n* = 2, 1.1%). There were some uterine causes like uterine fibroids, Asherman's syndrome and hysterectomy, and some ovarian causes like oophorectomy which caused infertility after the operation. The median interval between surgery and pregnancy was 20 months (range: 1–60 months).

**Table 2:** Pregnancy outcomes after bariatric surgery

Pregnancy outcomes variables	Number (%)
Pregnancy After Operation: - Group 1: miscarriage (abortion and stillbirth) - Group 2: completed	51 (28.8%) 18 (36.3%) 33 (64.7%)
Miscarriage:	
- Stillbirth - Abortion	1 (1.9%) 17 (33.3%)
In Vitro Fertilization (IVF) - Outcome: spontaneous abortion	2 2
Interval between operation and occurrence of pregnancy (months)	20 (1—60)
BMI on getting pregnant	31.9 (22.2—55.1)
%TWL at the time of pregnancy	33.5% (8.4–53.9%)
Weight gain during pregnancy (Kgs)	10.5 (0—47)
Complications During Pregnancy	36 (70.6%)
Type of complications:	
- Major - Minor	22 (61.1%) 14 (38.9%)

NB: * all variables expressed as median (min – max). BMI: body mass index, TWL: total weight loss

### Weight Loss Data at the Time of Pregnancy

The median BMI of 31.9 kg/m^2^ (range: 22.2–55.1 kg/m^2^) at pregnancy. The median %TWL in the whole cohort at the time of pregnancy was 33.5% (8.4–53.9%). According to the interval to pregnancy, the median %TWL in women who got pregnant during first, second and after 2 years from surgery were 28.6% (range: 8.4–44.2%), 34.4% (range: 12–53.6%), 35.1% (range: 16.6–53.9%), respectively. Recurrent weight gain occurred in 4 women (7.8%) who got pregnant after 2 years from surgery (27, 30, 36, and 36 months interval). Three women had % TWL > 20% at pregnancy as they conceived very early after surgery (1, 2, and 3 months interval).

### Pregnancy Complications

Pregnancy complications occurred in 70.6% of pregnancies (*n* = 36), most notably anemia, which affected (*n* = 28, 54.9%) of pregnancies and obstetric hemorrhage (*n* = 20, 39.2%). Twenty-two of pregnancy complications were major (*n* = 22, 61.1%) and required immediate intervention. Of 51 pregnancies, miscarriage occurred in 18 pregnant women (Group 1, 35.3%), among of which abortion occurred in 17 cases in the first and second trimester (*n* = 13, 72.2% and *n* = 4, 22.2%, respectively) and stillbirth occurred in 1 pregnancy (5.6%) in the second trimester. Hemorrhage was experienced in all cases of miscarriage, in the 1 st trimester (*n* = 13, 72.2%), in the 2nd trimester (*n* = 5, 27.8%), and no bleeding occurred in the 3rd trimester. Nine women suffered from anemia during pregnancy (50%) as shown in Table 3.

**Table 3:** Obstetric complications according to the pregnancy groups

Obstetric complications	Group 1 (*N* = 18)	Group 2 (*N *= 33)
Anemia During Pregnancy	9 (54.9%)	19 (57.6%)
Hemorrhage during pregnancy - Early Hemorrhage - Antepartum Hemorrhage	18 (100%) 15 (83.3%) 3 (16.7%)	2 (6.1%) 2 (6.1%) 0
Gestational Diabetes Mellitus	0	1 (3%)
Gestational Hypertension	0	1 (3%)
Time of delivery of Fetus		
- Term - Preterm	––	29 (87.9%) 4 (12.1%)
Method of Delivery		
- Vaginal - Cesarean section	––	4 (12.1%) 29 (87.9%)
Birth Weight of Neonate		
- Appropriate for gestational age (AGA) - Small for gestational age (SGA)	––	23 (69.7%) 10 (30.3%)
Neonatal Intensive Care Unit (NICU) Admission	––-	7 (21.2%)

In Table 3, complications in the group of women who completed the pregnancy (Group 2, *n* = 33) included maternal anemia (*n* = 19, 57.6%), GDM (*n* = 1, 3%), gestational HTN (*n* = 1, 3%), and early hemorrhage (*n* = 2, 6.1%) which were treated conservatively. No antepartum hemorrhage occurred in this group. Preterm births were encountered in 4 pregnancies (12.1%), and most deliveries were by cesarean section (*n* = 29, 87.9%). Regarding neonatal outcomes, 10 neonates (30.3%) were small for gestational age (SGA) and neonatal intensive care unit (NICU) admission was indicated for 7 neonates (21.2%). No congenital anomalies were encountered in any neonate.

### Pregnancy Outcomes Based on Time to Conception after Operation

Pregnancy outcomes were analyzed based on time to conception post-surgery: during the first year (*n* = 12), the second (*n* = 24) and after the second year (*n* = 15). During the first year interval, miscarriage (Group 1) occurred in 5 pregnant women and Group 2 in 7 pregnancies. Between 1–2 years, Group 1 (*n *= 6), and Group 2 (*n* = 18). After the 2nd year, Group 1 (*n *= 7), and Group 2 (*n* = 8). Stillbirth occurred during one pregnancy (6.7%) after the 2nd year (48 months interval). The abortion rate was highest in the first year and encountered in 5 cases (41.7%) and lowest in the second year (*n* = 6, 25%), with no significant difference between all groups (*p* = 0.490) as shown in Table 4. In general, pregnancy complications were most commonly experienced after the 2nd year (*n* = 13, 86.7%), among which anemia during pregnancy was the most prevalent complication after the 2nd year (*n* = 11, 73.3%). Regarding the neonatal outcome, preterm babies and small for gestational age occurred more often in the second year (*n* = 3, 12.5% and *n* = 6, 25%, respectively).

**Table 4:** Comparison of pregnancy outcomes based on time to conception after operation

Outcome	≤ 1 year (*N* = 12)	1 −2 years (*N* = 24)	< 2 years (*N* = 15)	P-value
Pregnancy outcome group:				
- Group 1 - Group 2	5 (41.7%) 7 (58.3%)	6 (25%) 18 (75%)	7 (46.7%) 8 (53.3%)	0.389
Stillbirth	0	0	1 (6.7%)	0.294
Abortion	5 (41.7%)	6 (25%)	6 (40%)	0.490
Complications During Pregnancy	9 (75%)	14 (58.3%)	13 (86.7%)	0.156
Anemia During Pregnancy	6 (50%)	11 (45.8%)	11 (73.3%)	0.226
Gestational Diabetes Mellitus	0	1 (4.2%)	0	0.563
Gestational Hypertension	0	0	1 (6.7%)	0.294
Hemorrhage				
- Early - APH	4 (33.3%) 1 (8.3%)	7 (29.2%) 0	6 (40%) 2 (13.3%)	0.383
Delivery of Fetus				
- At term - Preterm	7 (58.3%) 0	15 (62.5%) 3 (12.5%)	7 (46.7%) 1 (6.7%)	0.485
Birth weight of Neonate				
- AGA - SGA	5 (41.7%) 2 (16.7%)	12 (50%) 6 (25%)	6 (40%) 2 (13.3%)	0.669
Neonatal Intensive Care Unit	1 (8.3%)	4 (16.7%)	2 (13.3%)	0.658

Group1: miscarriage, Group 2: completed pregnancy. APH: anti partum hemorrhage, AGA: Appropriate for gestational age, SGA: Small for gestational age

## Discussion

One of the main obesity associated medical problems is female infertility which has a global concern [21, 22]. One of the most common mechanisms of female infertility is PCOS through insulin resistance, hormonal dysregulation, and hyperandrogenism [2]. In the present study, PCOS was encountered in 24 (13.6%) women and this incidence was relatively lower than previous studies [23, 24]. Moreover, 11 out of 12 women with infertility who got pregnant had PCOS in our study supporting prior literature indicating the beneficial impact of bariatric procedures on infertility associated with obesity [24].

This study evaluated the incidence of pregnancy following various types of bariatric surgeries. The overall post-surgical fertility rate in our study was 28.8%, which appears lower than that reported in previous studies [9, 25]. This discrepancy may be attributed to the fact that 44.6% (*n* = 79) of fertile women having completed their families and thus used contraception. Additionally, 9% of participants were still unmarried (potentially fertile) at the time of follow up, and cultural norms prohibit extramarital sexual activity. Among the women with infertility, however, the post-surgical fertility rate reached 75% (*n* = 12), aligning with findings by Deitel et al., who reported fertility rates as high as 88.9% in a similar population [25].

Although, bariatric surgery has significant impact on weight loss, it may have adverse effect on the pregnancy outcomes [26]. One of the important risks is malnutrition, micro and macronutrients deficiency, which has reported in previous studies [27, 28]. Important micronutrients that may decrease after bariatric surgery are iron, vitamin B12 and folate leading to maternal anemia and congenital anomalies of the fetus. In our study, the incidence of anemia was 54.9% in the pregnant women (50% in miscarriage group and 57.6% in completed pregnancy group) and it is higher than reported in the previous study of Devlieger et al. (20% and 40% at first trimester and delivery respectively) [29]. This difference may be contributed to that pregnant women in our study did not receive nutritional supplement regularly during pregnancy due to lack of planned follow up program.

Moreover, anemia emerged as a critical factor correlating with higher risks of maternal and fetal complications such as still birth, premature delivery, SGA, and maternal mortality. Anemia also decreases the tolerance of pregnant women with bleeding in the natal and postnatal period. We found that all women in the miscarriage group (*n* = 18) had experienced maternal bleeding (early and antepartum) and 50% had anemia leading to abortion or stillbirth reflecting the life threatening effect of anemia and other nutritional deficiency effect such as vitamin K deficiency during pregnancy [30, 31].

Gestational diabetes mellitus (GDM) (*n* = 1) and hypertension (HTN) (*n* = 1) occurred 2 pregnant women in the completed pregnancy group and no pre-eclampsia have encountered in the present study. Previous studies reported that the pregnant women after bariatric surgeries had lower risk of development of GDM and gestational HTN than pregnant obese women and still slightly higher than the incidence in our study [32]. In other hand, Wax et al., reported that rate of gestational HTN was higher in the bariatric surgery (gastric bypass) group than non-surgical control group and Patel el al, reported that there was no difference regarding the incidence of GDM between surgical and non-surgical control group [33, 34]

Miscarriage is common in the pregnant obese women than non-obese women and this may be attributed to the obesity related factors such as PCOS which is associated with high miscarriage rate [28]. Previous study reported decrease of miscarriage rate from 33.3% to 7.8% after bariatric surgery [35]. In contrast, Marceau et al., found that high miscarriage rate in pregnancies after biliopancreatic diversion [36] In the present study miscarriage occurred in 36.3% (*n* = 18) out of 51 pregnancies. This may attributed to that miscarriage is multifactorial natal adverse event which may be related to bariatric surgery itself due to nutritional deficiency, rapid weight loss and surgical complications and non-surgery related factors such as chromosomal abnormalities and poor quality of the ovulation.

We recorded a cesarean section (CS) rate of 56.7% following bariatric surgery. Christinajoice et al. reported that increase in rates of normal vaginal delivery rate of 73.7% while decrease in CS rate to 26.3% post-bariatric surgery [9]. Moreover, other study reported decrease in CS in bartiatric surgery group when compared to obese non-surgical group of pregnant women (45.9% and 65.8% respectively) [37]. The high CS rate in our study may be attributed to an increase in cesarean deliveries among Egyptian women as a common general practice, independent of body weight and previous bariatric surgeries.

Regarding to the neonatal outcomes, SGA rate was encountered in 10 pregnancies (30.3%) in the completed pregnancy group in the present study. Abdou et al. found that SGA incidence was 9.3% in the bariatric surgery group [12]. SGA is associated with degree of weight loss before pregnancy and failure of maternal weight gain and intra-uterine growth retardation (IUGR) [38]. Previous studies showed that the incidence of low fetal birth weight was significantly higher after the bariatric surgery especially the malabsorptive procedures [39, 40]. Moreover, Wax et al. and Patel et al. showed slightly lower rate of SGA than our study (26.3% and 26.9% respectively) [33, 34]. Higher SGA rate in the present study contributed to the poor nutritional status in the pregnant women. NICU admission was indicated in 7 pregnancies (21.2%) and it was higher than that reported in a previous study [14]

Preterm delivery was found in the present series in 4 pregnancies (12.1%), among of them 3 cases (12.5%) occurred in the 1–2 year interval and 1 case (6.7%) after 2 year interval from the bariatric surgery. Abdou et al. showed that the incidence of preterm delivery was 10% (*n* = 20) in the whole cohort with a predominance after the interval less than 1 year from the bariatric surgery (*n* = 8 out of 50, 16%) [12]. Similar rate was reported in Farghali et al. (*n* = 56, 10.3%) [14]. Higher rate of preterm delivery in our study resulted from poor nutritional status of pregnant women.

Regarding the timing of pregnancy after bariatric surgery, guidelines from the international societies recommend delay the pregnancy for at least 12–18 months after bariatric surgery to avoid the adverse effects of nutritional deficiencies during the weight loss phase [41, 42]. On the other hand, results from a retrospective study showed no maternal and fetal adverse events were occurred during pregnancy in the first 18 months [43]. Many factors should be taken into consideration for determination of the favorable timing of pregnancy including type of bariatric surgery, age of women seeking for conception, and the nutritional status after surgery [29]. Interestingly, our findings diverge from those of earlier studies which reported no significant difference between the interval between bariatric surgery and conception and pregnancy complications with higher complication rate beyond two years (86.7%). Previous study highlighted increased risk during the first 12 months post-surgery [12].

Finally, the overall pregnancy related complications were higher than previous studies due to the limited attention to nutritional status in the follow-up care, where the primary focus remained on weight loss before pregnancy and lack of adequate multidisciplinary follow-up and specific prenatal program for women after bariatric surgery which increases the risk of gestational complications and unfavorable pregnancy outcomes.

## Limitations of the Study

This study was conducted in a single center and so the results may be influenced by specific patients’ demographics, surgical techniques, and postoperative care protocols limited to our institution. Relatively small number of women who got pregnant, so the results should be interpreted with caution. It was a retrospective study which may lack some data and introduce bias affecting the reliability of conclusions. We did not assess hormonal profiles as the hormonal imbalance is critical factor affecting the fertility and pregnancy outcomes. We did not conduct comprehensive nutritional evaluations for detection of nutritional deficiencies which are common after bariatric surgery and significantly impact the maternal and fetal health. Finally, there is no specific follow up program protocol for women who get pregnant after bariatric surgery, thus it lacks standardization for the care of this groups of patients.

## Conclusions

Bariatric surgery may improve the fertility rates. However, pregnancies after surgery may result in potential complications particularly if the pregnancy occurs early during the first year and delayed after 2 years from surgery. Lack of adequate multidisciplinary follow-up and specific prenatal program for women after bariatric surgery increases the risk of gestational complications and unfavorable pregnancy outcomes. Thorough nutritional evaluation prior to conception through specific follow up program in this group of women is essential to avoid related maternal and fetal complications.

## Supplementary Information

Below is the link to the electronic supplementary material.

Supplementary file1 (PPTX 793 KB)

## Data Availability

No datasets were generated or analysed during the current study.
