# Supplementary material for: Does Bariatric Surgery Affect Pregnancy in Women in the Childbearing Period? A Retrospective Single Center Study
Source: Obes Surg. 2025 Aug 29;35(9):3877–87. doi: 10.1007/s11695-025-08197-6 (PMC12457470; doi:10.1007/s11695-025-08197-6)
Supplement: Supplementary file 1 — Supplementary file1 (PPTX 793 KB) [file 11695_2025_8197_MOESM1_ESM.pptx]

## Slide 1
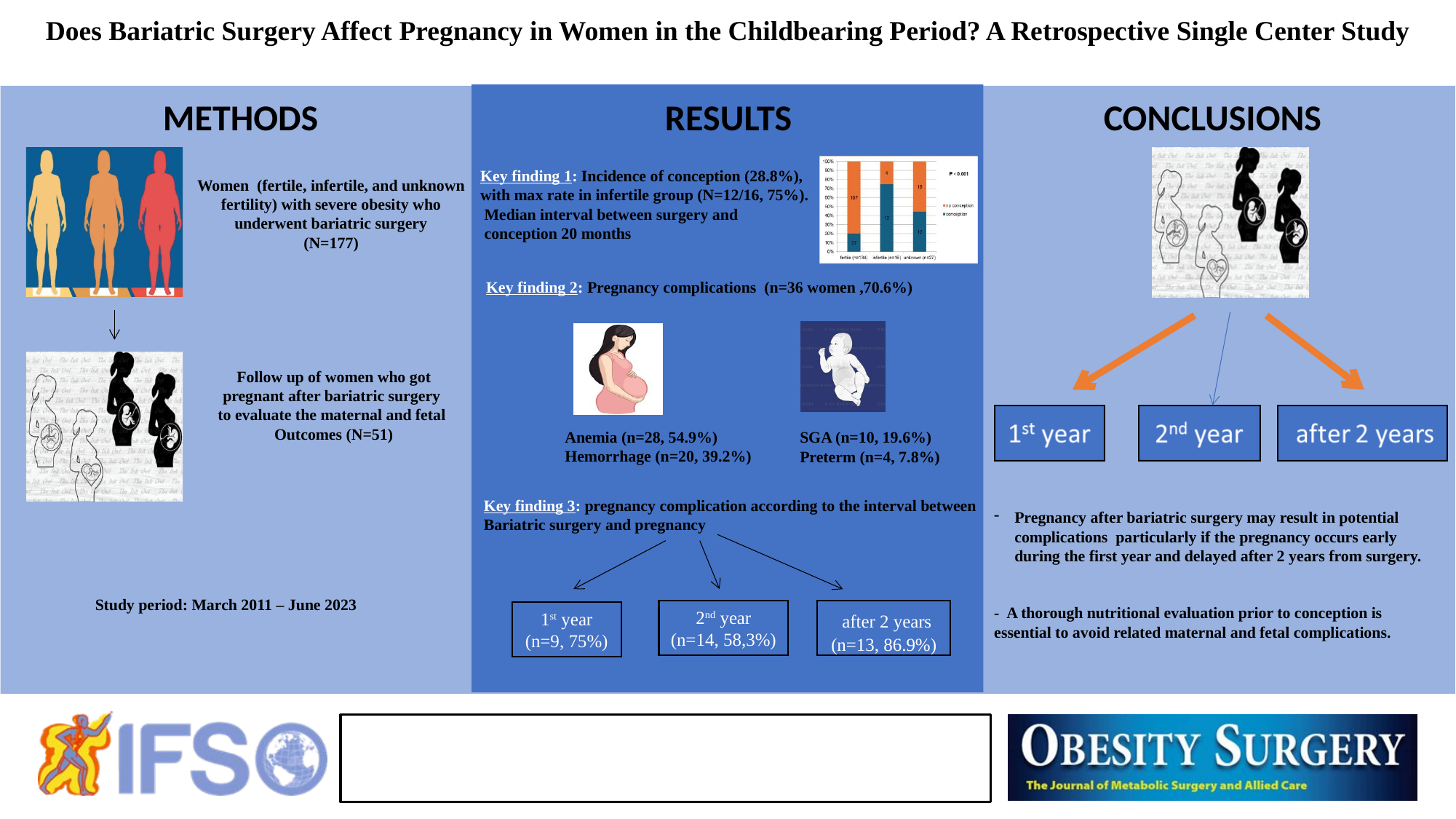

Does Bariatric Surgery Affect Pregnancy in Women in the Childbearing Period? A Retrospective Single Center Study
Conclusion
 METHODS
RESULTS
CONCLUSIONS
Key finding 1: Incidence of conception (28.8%),
with max rate in infertile group (N=12/16, 75%).
 Median interval between surgery and
 conception 20 months
Women (fertile, infertile, and unknown
 fertility) with severe obesity who
underwent bariatric surgery
(N=177)
Key finding 2: Pregnancy complications (n=36 women ,70.6%)
Follow up of women who got pregnant after bariatric surgery
to evaluate the maternal and fetal
Outcomes (N=51)
Anemia (n=28, 54.9%)
Hemorrhage (n=20, 39.2%)
SGA (n=10, 19.6%)
Preterm (n=4, 7.8%)
Key finding 3: pregnancy complication according to the interval between
Bariatric surgery and pregnancy
Pregnancy after bariatric surgery may result in potential complications particularly if the pregnancy occurs early during the first year and delayed after 2 years from surgery.
- A thorough nutritional evaluation prior to conception is essential to avoid related maternal and fetal complications.
Study period: March 2011 – June 2023
2nd year
(n=14, 58,3%)
 after 2 years
(n=13, 86.9%)
1st year (n=9, 75%)
